# Hepatitis E Virus Infection: Neurological Manifestations and Pathophysiology

**DOI:** 10.3390/pathogens10121582

**Published:** 2021-12-03

**Authors:** Sébastien Lhomme, Florence Abravanel, Pascal Cintas, Jacques Izopet

**Affiliations:** 1 Infinity, Université Toulouse, CNRS, INSERM, UPS, 31300 Toulouse, France; abravanel.f@chu-toulouse.fr (F.A.); izopet.j@chu-toulouse.fr (J.I.); 2 Laboratoire de Virologie, Hôpital Purpan, CHU Toulouse, 31300 Toulouse, France; 3 Service de Neurologie, Hôpital Purpan, CHU Toulouse, 31300 Toulouse, France; cintas.p@chu-toulouse.fr

**Keywords:** hepatitis E virus, neurological manifestations, extra-hepatic manifestations

## Abstract

Hepatitis E virus (HEV) is the first cause of viral hepatitis in the world. While the water-borne HEV genotypes 1 and 2 are found in developing countries, HEV genotypes 3 and 4 are endemic in developed countries due to the existence of animal reservoirs, especially swine. An HEV infection produces many extra-hepatic manifestations in addition to liver symptoms, especially neurological disorders. The most common are neuralgic amyotrophy or Parsonage–Turner syndrome, Guillain–Barré syndrome, myelitis, and encephalitis. The pathophysiology of the neurological injuries due to HEV remains uncertain. The immune response to the virus probably plays a role, but direct virus neurotropism could also contribute to the pathophysiology. This review describes the main neurological manifestations and their possible pathogenic mechanisms.

## 1. Introduction

The hepatitis E virus (HEV) is a leading cause of acute hepatitis worldwide, causing 2 million infections and 70,000 deaths every year [1]. HEV is a single-strand, positive sense RNA virus with an icosahedral capsid. The 7.2-kb long genome is capped at the 5′ end and polyadenylated at the 3′ end. It contains three main open-reading frames (ORF), ORF1, ORF2, and ORF3 [2]. ORF1 encodes a non-structural protein with enzymatic domains, including a methyltransferase, a papain-like cysteine protease, a helicase, and a RNA-dependent RNA polymerase. It also contains domains like the Y domain, the polyproline region (PPR), and a macro domain, whose functions are unknown. ORF2 encodes the capsid protein that exists in at least two forms. The secreted form (ORF2s) is an N-glycosylated and sialylated dimer. It has a signal peptide and is a different translation product from the infectious form (ORF2i), the actual capsid protein, whose translation is initiated at a previously unrecognized internal AUG codon [3]. ORF3 encodes a small phosphoprotein involved in virus egress and the apical release of HEV [4,5]. The ORF3 protein can also act as an ion channel [6]. ORF4 has been described in HEV1 [7]. It encodes a protein that is translated following endoplasmic reticulum stress to increase the activity of the HEV polymerase.

HEV is the only member of the *Hepeviridae* family, which is composed of two genera: *Piscihepevirus* (cutthroat trout virus) and *Orthohepevirus* (mammalian and avian strains), composed of four species (A–D) [8,9]. Most human infections are due to *Orthohepevirus A*, which can also infect other mammals. *Orthohepevirus B* infects chickens; *Orthohepevirus C* infects rats and ferrets, and *Orthohepevirus D* infects bats. *Orthohepevirus A* is the largest species, with at least eight distinct HEV genotypes that infect human (HEV1, 2, 3, 4, and 7), pigs (HEV3 and 4), wild boar (HEV3, 4, 5, and 6), rabbits (HEV3), deer (HEV3), mongooses (HEV3), yaks (HEV4), and camels (HEV7 and HEV8).

HEV1 and HEV2 circulate in developing countries where they only infect humans. HEV3 is widely distributed around the world, while HEV4 is found mainly in Asia. The HEV3 and HEV4 genotypes are mainly transmitted zoonotically via animal reservoirs, such as pigs, wild boar, deer, and mongooses [10]. There have been recent reports of nine cases of patients infected with *Orthohepevirus C* HEV [11,12,13], which is surprising because this virus differs genetically from other human pathogenic strains [14].

HEV infections are usually asymptomatic [15,16,17]. Symptoms generally last just a few weeks in most patients. The classic acute icteric hepatitis presents first with a prodromal phase with non-specific symptoms (nausea, vomiting, fever, etc.) followed by the icteric phase, characterized by jaundice and dark urine [18,19]. HEV infections are most of the time self-limiting in immunocompetent patients [20], but chronic infection, defined by the persistence of the virus for more than three months [21], can occur in immunocompromised patients, including solid organ transplant recipients [22,23,24], patients with hematological disease receiving chemotherapy [25,26,27], those given stem cell transplants [28], those co-infected with HIV with a low T CD4 + count (<200/mm^3^) [29,30], and patients with rheumatic disorders on heavy immunosuppression immunotherapy [31,32]. Extra-hepatic, especially neurological, manifestations have frequently been described in both acutely and chronically HEV infected patients.

This review describes these clinical manifestations and discusses the pathogenic mechanisms that might be involved.

## 2. Clinical Manifestations

**Figure 1:**
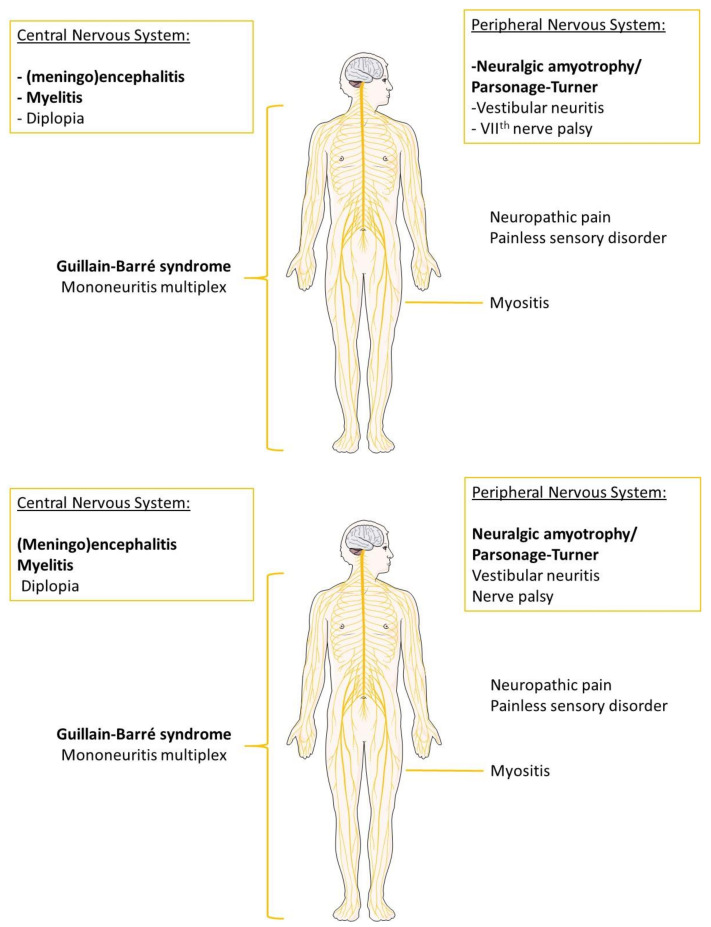
Main neurological symptoms associated with an HEV infection. The most frequent manifestations are in bold. Other manifestations, including neuritis, Bell’s palsy, neuropathic pain, painless sensory disorders (numbness, tingling), diplopia, or myositis, were described in few patients.

The possibility of neurological manifestations following HEV infection was first suspected in 2000, when an Indian patient developed Guillain–Barré syndrome (GBS) following an HEV infection [33]. Then, in 2009, a 53 year old patient with a bilateral neuralgic amyotrophy (NA) showed an isolated abnormal liver enzyme profile together with anti-HEV IgM antibodies [34]. The HEV genome was detected in a Thai patient suffering from NA in 2010 [35]. At about the same time, HEV RNA was detected in the cerebrospinal fluid (CSF) of a kidney-transplant recipient chronically infected with HEV and presenting neurological symptoms [36]. Since then, hundreds of HEV-infected patients with neurological manifestations have been described. Some retrospective studies found the incidence of neurological symptoms in HEV-infected patients to be 5.5–7.2% [37,38], while others report that 16.5–30% of HEV infected patients develop neurological symptoms [39,40]. These patients are usually viremic when neurological symptoms appear. Most of the cases have been HEV3-infected patients in Europe or HEV1-infected patients in developing countries. Asian cases are not as well documented as European ones probably because HEV is not systematically genotyped. A Chinese retrospective study found no association between HEV4 and neurological manifestations [41], while a Japanese patient infected with an HEV4 strain was reported to have suffered bilateral facial paralysis [42]. The best-documented manifestations are NA or Parsonage–Turner syndrome, GBS, encephalitis, and myelitis [43] (Figure 1). Almost all the HEV-infected patients presenting with neurological symptoms had normal to mildly abnormal liver function and most did not present jaundice.

### 2.1. Neuralgic Amyotrophy

Neuralgic amyotrophy (NA), also known as Parsonage–Turner syndrome or brachial plexus neuritis, is characterized by severe neuropathic pain in the arms and shoulders, followed by patchy weakness, atrophy, and sensory disturbance [44]. The disease usually involves the brachial plexus. NA is considered to be a post-infection, immune-mediated neuropathy.

The various studies of HEV infections in patients with NA have almost all been done on HEV3-infected Europeans. A multicentric study involving four centers in France, the UK, and the Netherland prospectively tested over 450 consecutive patients with acute-onset non-traumatic neurological injury between February 2015 and March 2016. This study found that 11/464 (2.4%) of them were positive for anti-HEV IgM ± HEV RNA [43]. The three cases of NA were described in patients infected with HEV. Similarly, retrospective HEV testing of nine Cornish patients with NA (2011–2013) and a prospective testing of 38 consecutive Dutch patients with NA (2004–2007) found that 5/47 (10.6%) patients with NA showed evidence of an HEV infection at the onset of their illness. Anti-HEV IgM were detected in all five patients and HEV RNA in 4/5 [45]. A Swiss study found that 6/93 (6.5%) patients with an HEV infection, based on RNA detection, had NA [46]. Lastly, HEV RNA and the intrathecal synthesis of anti-HEV IgM were observed in a patient with NA, suggesting an HEV infection in the central nervous system (CNS) [47]. A multicentric study involving 11 centers from seven European countries found that patients with HEV-associated NA had a clinical phenotype that was distinct from that of patients with NA without any evidence of an infection with HEV. Patients with HEV-associated NA were significantly more likely to have bilateral involvement (80%) than were non-HEV patients with NA (9%) [48]. A French study also reported that four patients suffering from NA had bilateral involvement associated with an HEV infection [49]. Damage outside the brachial plexus, including the phrenic nerve and lumbosacral plexus injury, were also more frequent in HEV-infected patients (59%) than in HEV-free patients (11%) [48]. However, despite these differences, the outcomes in both groups were similar.

### 2.2. Guillain–Barré Syndrome

Guillain–Barré syndrome (GBS) is the most common cause of acute neuromuscular paralysis in countries where poliomyelitis has been eliminated [50]. GBS is diagnosed based on clinical characteristics and biological criteria nearly a century after the eponymous publication of Georges Charles Guillain and Jean-Alexandre Barré [51]. GBS is a subacute disorder of the peripheral nerves and nerve roots that leads to rapidly progressing limb weakness and sensory deficit; this can progress to respiratory failure in the most severe cases. Liver function is abnormal in 10–40% of patients [52,53] and hyponatremia in 25% [54].

Three recent case-control studies performed in Bangladesh [55], Japan [56], and the Netherlands [57] support the association of an HEV infection with GBS. The Bangladeshi prospective case-control study on 100 consecutive GBS cases enrolled between July 2006 and June 2007 used two controls per case. Anti-HEV-IgM antibodies were more frequently detected in patients with GBS (11%) than in patients with other neurological symptoms (2%; *p* < 0.01) [55]. The Japanese study examined sera from 63 Japanese patients with GBS or Miller Fisher syndrome (MFS, a variant of GBS that is characterized by a triad of ataxia, ophthalmoplegia, and areflexia [58,59]) and 60 controls matched for age and sex collected between 1998 and 2014. All the samples were tested for both anti-HEV IgM and IgG. Only 3/63 patients had both anti-HEV IgM and high hepatic enzyme activities: two suffered from GBS and one from MFS. None of the control-group patients was anti-HEV IgM positive, suggesting that 3.2% of patients with GBS had an acute HEV infection [56]. The Dutch study determined the frequency of HEV infection in 201 patients with GBS and 201 healthy controls by anti-HEV serology. Anti-HEV IgM antibodies were detected in 10 GBS patients and one control patient. Thus, 5% of patients with GBS had an acute HEV infection [57]. Lastly, a Belgian study that analyzed 63 samples collected between 1 January 2007 and 1 November 2015 showed that 8% (6/73) of Belgian patients with GBS had anti-HEV IgM antibodies, indicating an HEV infection [60]. One limitation of the studies investigating the presence of only anti-HEV IgM (without the determination of HEV RNA) is the possibility of false-positive results in the context of autoimmunity. Conversely, false negative results may not be excluded [61,62].

### 2.3. Other Neurological Manifestations

HEV-infected patients may also suffer from other manifestations, such as neuropathic pain that can evoke small fiber neuropathy, painless sensory disorders like numbness or tingling, encephalitis/myelitis, diplopia, mononeuritis multiplex, vestibular neuritis, myositis, and peripheral neuropathy [39,63]. In these patients, the liver function is usually normal or mildly abnormal, suggesting that the clinical picture is dominated by these neurological symptoms.

Meningoencephalitis has been described in a few patients [41,43,64,65,66] but HEV RNA was not always detected in their CSF. One patient was diagnosed by metagenomics next-generation sequencing following CSF analysis. This result prompted the physicians to test for anti-HEV IgM; this was positive, as was the plasma HEV viremia (5.96 × 10^6^ IU/mL) [64]. One kidney transplant patient was found to have meningoencephalitis due to *Orthohepevirus C*. The concentration of rat HEV-C1 RNA in the CSF was 1.65 × 10^3^ copies/mL [11].

Anicteric patients can also suffer from cerebral ischemia [41,43]. A woman who developed reduced lower limb power, brisk reflexes, extensor plantars, a sensory level at T8, and reduced anal sphincter tone was clinically diagnosed as having transverse myelitis [67]. HEV RNA was found in both the blood and CSF of this anicteric patient.

A group of six French patients infected with HEV3 was found to have mononeuritis multiplex, defined by asymmetric, asynchronous involvement of the non-contiguous nerve trunks [68]. They had all suffered from neuropathic pain and paresthesia in one or more nerve segments with hyporeflexia or areflexia. Five of them were also suffering from peripheral neuropathy and small-fiber neuropathy [41,69,70].

Cranial neuropathies have been associated with an HEV infection, including isolated facial nerve palsy (Bell’s palsy) and involvement of the vestibular nerve [42,43,69,71,72]. Other HEV-infected patients have been found to have myasthenia gravis [49] or myositis [73]. Lastly, a few patients with an acute HEV infection were found to be suffering from meningoradiculitis, involving both the meninges and nerve roots, with the lumbosacral region being the most common site affected [68,74,75]. Another acutely HEV-infected patient recently presented with severe neurological deficits and evidence of multiple disseminated inflammatory lesions of the CNS, including musculoskeletal weakness, bladder and bowel retention, blurred vision, and ascending hypoesthesia up to the level of T8 [76].

## 3. Pathophysiology

The pathophysiology of the neurological injuries due to HEV remains uncertain. The immune response following virus infection is probably involved. One argument in favor of immune system involvement is the greater frequency of neurological injuries in immunocompetent patients (31/137; 22.6%) than in immunocompromised ones (2/63; 3.2%, *p* < 0.001) [39]. The six patients suffering from NA and one from GBS were all immunocompetent, suggesting that these manifestations are immune-mediated, perhaps due to molecular mimicry. In another study, all the 15 patients suffering from NA were immunocompetent [40]. Lastly, anti-ganglioside GM1 and GM2 antibodies were found in patients infected with HEV [77,78,79]. This hypothesis is in line with the mechanisms currently thought to be responsible for NA and GBS pathophysiology.

Direct virus neurotropism could also explain these neurological disorders. A kidney transplant patient chronically infected with HEV presented a pyramidal syndrome. Simultaneous analysis of the HEV quasi-species in the cerebrospinal fluid and the serum showed that the two populations were different, suggesting that HEV was replicating in the CNS [36]. A recent study suggested that the compartmentalization of HEV in the CNS requires a prolonged infection [80]. A more recent report described an immunocompetent patient who developed a chronic HEV infection and suffered from hyperesthesia of the arms and legs. Ribavirin therapy cleared the virus in the plasma and the feces, but HEV RNA persisted in the CSF for more than a year despite the therapy [81]. There was also evidence of intrathecal anti-HEV IgG synthesis. The sequence of the PPR of the virus isolated from the CSF had eight amino acid deletion and thus was shorter than the virus in the serum, suggesting that the CNS can support independent HEV replication [81].

### 3.1. In Vivo Evidence of the CNS Tropism of HEV

Intraperitoneal infection of Mongolian gerbils with an HEV4 strain (Genbank number KMN024042) isolated from a pig liver led to the detection of both positive and negative RNA strands and ORF2 protein in the brains and spinal cords of the gerbils, indicating that HEV replicates in these tissues. In addition, the reduction of the expression of zonula occludens-1 (Zo1) suggests that HEV4 damages the blood-brain barrier in order to enter the CNS. Pathological changes were also reported, including degeneration and necrosis of neurons, microglia nodules, Purkinje cell necrosis, and infiltration by inflammatory cells [82]. These features are quite similar to those reported in humans. Rabbits intraperitoneally infected with a porcine HEV4 strain (Genbank number KJ123761) from intestinal content showed similar features. Both positive and negative RNA strands were detected in the brain and spinal cord of these rabbits. ORF2 antigen was also detected immunohistochemically in their brains and spinal cords, mainly in neural cells and perivascular areas [83]. Lastly, mice and rhesus macaques were intravenously injected with an HEV4 strain (KM01) isolated from porcine feces [84]. HEV RNA was detected in their brain tissues, and HEV ORF2 protein was detected immunohistochemically in the granular layer of the cerebellum. All these experiments confirm that HEV can infect the brain of several animal species.

### 3.2. In-Vitro Evidence of the CNS Tropism of HEV

A replicon system based on the HEV3 Kernow-C1 p6 strain, in which ORF2 is replaced by the *Gaussia* luciferase and is secreted following RNA replication [85], was used to study the capacity of human neural cell lines to support HEV replication. Glioblastoma multiforme (DBRTG), desmoplastic cerebellar medulloblastoma (DAOY), glioblastoma astrocytoma (U-373 MG), and oligodendrocytic (MO3.13) cell lines all supported HEV replication but with differing efficiencies; MO3.13 was the most efficient [86]. The oligodendrocytic cell line MO3.13 supported the infection, replication, and release of HEV [86]. Oligodendrocytes are important for the development of central myelin. GBS is accompanied by damage to the myelin of peripheral neurons [87]. Peripheral nerves are also believed to be damaged in these infections that induce a cross-reacting immune response targeting axolemma or Schwann cell antigens [87]. Others have reported that HEV can efficiently infect human neural cells, neuroblastoma SH-SH5Y, neuroepithelioma SK-N-MC, and glioblastoma U87 and U343 cells in vitro [84]. Ribavirin and/or IFN-alpha decreased HEV replication in these systems [84,86].

Human pluripotent stem cells that have differentiated into mesodermal and neuroprogenitors cells cannot be infected by the Kernow-C1 p6 strain, but transfection of these cells with a subgenomic replicon system (Kernow-C1 p6/luc) allows HEV replication [88]. Conversely, Zhou et al. reported that induced pluripotent stem cell-derived human neurons can be infected with the Kernow-C1 p6 strain [84]. The same group used the Kernow C1 p6 strain to show that primary cultures of mouse neurons support the complete HEV lifecycle [84]. Lastly, changes in the tight-junction proteins (including claudin 5, occludin and zonula occludens-1) were described in vitro by using primary cultures of human brain microvascular cells (HBMVCs) infected with an HEV4 strain for 48 h [83]. This suggests that HEV enters the CNS by breaking the blood-brain barrier.

## 4. Diagnosis and Treatment

An HEV infection is usually diagnosed using a combination of serology and molecular tests [89,90]. HEV RNA becomes detectable in the blood and stools during the incubation period and can persist for four weeks in the blood and for six weeks in the feces [91]. Anti-HEV IgM antibodies are detectable at the same time as liver enzyme activities increase [92]. An anti-HEV IgM test is done first if an HEV infection is suspected due to the good performances of the assays available [90]. HEV RNA testing is essential for diagnosing infections in immunocompromised patients because anti-HEV IgM antibodies may be absent due to immunosuppression. Patients with evidence of neurological manifestations, especially NA and GBS, should be tested for HEV.

Ribavirin is presently the treatment of choice for patients with a chronic HEV infection [89]. The first study reporting an antiviral effect of ribavirin included two patients [93]. The same year, another single center study showed its antiviral effect on six patients [94]. A French multicenter study showed that after ribavirin monotherapy for a median duration of three months with a median dose of 6000 mg per day, a sustained virologic response (SVR) was observed in 46/59 (78%) of the patients [95]. Retreatment of 6/10 patients with ribavirin treatment failure increased the SVR rate to 50/59 (84.7%). These results were then confirmed in a large European multicenter study cohort including 255 patients from 30 centers [96]. After ribavirin monotherapy for a median duration of three months with a median dose of 6000 mg per day, a SVR was obtained in 207/255 (81.2%) patients. The SVR rate even rose to 229/255 (89.8%) when a second treatment with a longer course was offered in 36/48 patients with first ribavirin treatment failure [96]. It has been proposed for treating severe acute hepatitis [89,97], but this needs to be confirmed. There is, as yet, no evidence for using ribavirin to treat HEV-associated neurological disorders despite its efficiency in vitro [84,86]. Three patients (two with NA and one with mononeuritis complex) were treated with ribavirin [68], but its effect on the evolution of neurologic symptoms, especially for patients with NA, needs further investigations.

## 5. Conclusions

The neurological manifestations due to HEV are now well recognized. NA and GBS are the most frequent, followed by meningo-encephalitis. These three clinical pictures are causally associated with HEV infections. HEV must be considered in the differential diagnosis of idiopathic neurological disorders, including those of patients with normal liver function. The recent finding of HEV RNA persisting in the CSF despite its clearance from the blood and stool should lead physicians to look for HEV RNA in the cerebrospinal fluid of patients with an HEV infection if neurological symptoms persist. The pathophysiology of such manifestations remains to be determined. Similarly, the role of ribavirin in these situations also needs careful evaluation.

## Data Availability

Not applicable.
